# Return to Skiing After Proximal Tibial Fracture: Postoperative Reality and Initial Expectations

**DOI:** 10.3390/jcm13237352

**Published:** 2024-12-02

**Authors:** Lena Keppler, Fanny Navarre, Alexander Martin Keppler, Fabian Maria Stuby, Wolfgang Böcker, Tim Saier

**Affiliations:** 1 Department of Orthopaedic and Trauma Surgery, Musculoskeletal University Center Munich (MUM), LMU Hospital Munich, 80336 Munich, Germany; lena.keppler@med.uni-muenchen.de (L.K.);; 2 Department of Trauma Surgery, BG Trauma Center Murnau, 82418 Murnau, Germany; 3 Orthopädisches Versorgungszentrum, München Innenstadt, 80331 Munich, Germany

**Keywords:** tibial fracture, skiing, return to ski, ORIF, outcome, functionality, quality of life, expectation, fulfillment

## Abstract

**Background/Objectives:** The aim of this study was to investigate patient-reported outcomes of patient expectations and fulfillment of expectations in alpine skiers who had a skiing accident and suffered a complex proximal tibial fracture (AO/OTA—Type B or C) which was treated surgically with open reduction and internal fixation. **Methods:** In this prospective study, 38 consecutive patients who suffered a complex tibial fracture (AO/OTA—Type B and C) caused by a skiing accident were evaluated. Before surgical treatment with open reduction and internal fixation, patient expectations were evaluated regarding outcomes on knee functionality (e.g., pain) and the return to skiing. At follow-up ≥ 1 year after surgery, an individualized questionnaire was used to evaluate whether their preoperatively formulated expectations had been fulfilled (rated 0–2). In addition, the Knee injury and Osteoarthritis Outcome Score (KOOS), and SF-12 was used. **Results:** Preoperatively, 76% (n = 29) of patients stated that it was “not so important” to be able to return to their initial skiing level, 50% (n = 19) of patients did not expect to be able to ski again, 34% (n = 13) expected to return to skiing at a significantly lower level, and 16% (n = 6) expected to return to skiing with minor restrictions at most. Postoperatively, the return to skiing rate on initial level was 32% (n = 12); 50% (n = 19) stated that their initial skiing level was not reached again but they were able to ski with moderate restrictions, 10% (n = 4) patients stated that no return to skiing was possible, 50% (n = 19) stated that their expectations were fully met, and n = 7 (18%) stated that their preoperative expectations were not met at all. The mean SF-12 physical component score (PCS) was 52, and the mean mental component score (MCS) was 49.9. The mean Numeric Rating Scale (NRS) was 2.3. The mean KOOS for pain was 86.1 (SD 17.1), for symptoms 62.2 (SD 12.9), for ADL 90.7 (SD 14.4), for sports 74.2 (SD 25.7), and for quality of life (QOL) 66.6 (21.0). **Conclusions:** After suffering from a complex proximal tibial fracture (AO/OTA—Type B or C) in a skiing accident, preoperative patient expectations to return to skiing is limited. This patient-reported outcome of patient expectations was confirmed by the findings of the patient-reported fulfillment of expectations at least one year after surgery as only 32% of patients returned to their initial skiing level, 50% returned to skiing with limitations, and 50% of patients did not meet their preoperative expectations to return to alpine skiing. The results of this study emphasize the importance of directing patients towards realistic expectations by managing the patients’ understanding of the severity of their injury and realistic outcomes, and providing realistic forecasts of postoperative outcomes.

## 1. Introduction

Tibial plateau fractures are a common injury that has seen an increase in incidence over time [1]. Regarding trauma mechanism, proximal tibial plateau fractures may be caused by low- or high-energy trauma. One relevant high-energy trauma mechanism is alpine skiing, and therefore it may be responsible for a high number of cases worldwide depending on different areas [2,3,4].

In young and active individuals, these fractures can affect their capacity to engage in sports, as they may not only involve substantial joint damage in the short term but may also lead to long-term complications such as residual pain, limited knee function, the development of secondary osteoarthritis, and even a need for total knee replacement [5,6]. The existing literature evaluating functional outcomes and the return to sports after operatively treated proximal tibial plateau fractures demonstrated different rates of returning to an active lifestyle and returning to sports, depending on different factors such as trauma mechanism or the severity of the injury [7,8].

Returning to high-impact sports appears to be difficult or even impossible in the case of complex joint injury due to the primary damage of the joint and the development of secondary degenerative alterations leading to osteoarthritis [9,10]. In patients who have undergone operative treatment for intra-articular knee fractures, the incidence of post-traumatic osteoarthritis (OA) ranges from 21% to 75% [11,12]. This figure represents the proportion of patients who develop OA despite the achievement of adequate reduction and the stable fixation of the fracture. The risk factors identified for the development of secondary OA include advanced age and osteoporotic bone quality, the presence of comorbidities, the malalignment of the knee, articular collapse, and fracture pattern [13,14]. Statistics from 2021 assume that around 112 million people worldwide ski [15]. Tibial plateau fractures currently play an important role in skiing accidents.

The risk of injury when skiing has been steadily increasing for some years now and a shift from lower leg injuries towards knee injuries has been found [16,17]. According to data from Germany in the 2022/2023 skiing season, the number of injured skiers will increase significantly and is projected to be around 42,000 to 44,000 skiers [18]. Even the risk of hospitalization after a skiing accident has also risen from 1.6 to 1.73 per 1000 skiers. In these accidents, the knee joint is still the most frequently injured part of the body with an incidence of 26% in Germany [18]. In recent years, an increase in severe bony proximal tibia fractures has been observed [2,19,20,21]. Thus, proximal tibial plateau fractures currently play an important role in skiing accidents. Several previous studies have specifically investigated the issue of returning to skiing after tibial plateau fractures [10,22,23]. These studies have demonstrated rates of returning to skiing between 53% and 87% [1,23,24]. Following surgical intervention with open reduction and internal fixation for proximal tibial plateau fracture, it is possible that returning to skiing may present a greater challenge than other desired recreational activities, given the significant forces across the joint required for skiing.

To be able to return to high-impact sports such as alpine skiing, good knee functionality (e.g., range of motion and joint stability) is essential. Previous studies have already shown the functional outcome after proximal tibial fractures, which may be unsatisfying [1,24].

However, in the existing literature, to date, there has been a lack of measurement of patient-reported outcome measures evaluating patients’ expectations of returning to alpine skiing and the fulfillment of patients’ expectations. This is an important measurement to evaluate the patients’ understanding of the severity of their injuries. With regard to this, it has already been shown in other studies that patients often underestimate the severity of their injury, and patients’ expectations of the surgical outcome may overestimate realistic results, which may lead to dissatisfaction with the outcome [25]. Furthermore, the fulfillment of preoperatively formulated expectations of skiers regarding the outcome of surgery for complex proximal tibial fractures has not yet been investigated.

This study aimed to evaluate (1) patients’ preoperative expectations to return to skiing, (2) the postoperative fulfilment of their expectations, and (3) knee functionality.

## 2. Material and Methods

This prospective study was conducted at a Level-1 trauma center close to several skiing areas. Between 12/2017 and 12/2019, 38 consecutive patients who suffered from a complex proximal tibial fracture (AO/OTA type B or C) caused by an alpine skiing accident were included in this study. All patients were indicated for surgical treatment with open reduction and internal fixation (ORIF). Written consent to participate in this study was obtained from each patient. The mean follow-up time was 3.9 years.

### 2.1. Preoperative Evaluation of Patient Expectations to Return to Alpine Skiing

Following a clinical and radiological diagnosis of a proximal tibial fracture, AO/OTA Type B or C surveys regarding patients’ expectations were handed out after written surgical consent was obtained. The survey was completed between 24 h and 48 h prior to surgery. Patients were evaluated on the following question: How important is it to you to return to your initial sporting level? Items were rated on a four-point Likert scale ranging from 1 (very important) to 4 (not important). Further, we evaluated their expectations to return to alpine skiing. Patients could indicate whether they expected to return to their initial skill level with no, minor, or major restrictions. In addition, they were asked if they expected to return to alpine skiing at a slightly lower level, return at a significantly lower level, or not to return at all.

### 2.2. Postoperative Evaluation of Fulfillment of Patient Expectations to Return to Alpine Skiing

At least 12 months postoperatively, each patient received an individual questionnaire regarding the fulfillment of their preoperatively formulated expectations. The degree of fulfillment of expectations depended on whether expectations were not met at all, expectations were partially met, or expectations were fully met. Additionally, the Knee Injury and Osteoarthritis Outcome Score (KOOS) and the SF-12 questionnaire (with a physical component score (PCS) and a mental component score (MCS)) were evaluated. Pain was measured via the Numeric Rating Scale at a range of 0–10.

### 2.3. Statistical Analysis

For the purposes of statistical analysis, IBM SPSS Statistics version 29.0 (IBM Corp., Armonk, NY, USA) was employed. In order to analyze the demographic data, the mean and standard deviation (SD) were calculated. The data were tested for normal distribution, and a parametric test (*t*-test) was employed. To demonstrate the existence of a correlation, Spearman’s rho (r) was employed. The level of significance was set at *p* ≤ 0.05. A power analysis was not conducted due to a lack of comparative data.

## 3. Results

In total, 38 patients were evaluated at final follow-up (100% follow-up rate); of these, 17 (44.7%) were male and 21 were female. The mean age at trauma was 45.7 years (min.: 18 years; max.: 72 years; SD 13.2). In total, 18 (47.4%) patients suffered an AO-B fracture, and 20 (52.6%) suffered an AO-C fracture. The mean BMI was 24.1 kg/m^2^ (SD 4.6). In total, 33 (86.8%) stated that they are regular skiers, 4 (10.5%) did not ski regularly, and 1 was a professional skiing athlete.

Preoperatively, 29 (76.3%) patients stated that it was “not so important” to be able to return to their initial alpine skiing level, and 5 patients (13.1%) stated that returning to their initial alpine skiing level was “very important” or “important” to them.

**Figure 1:**
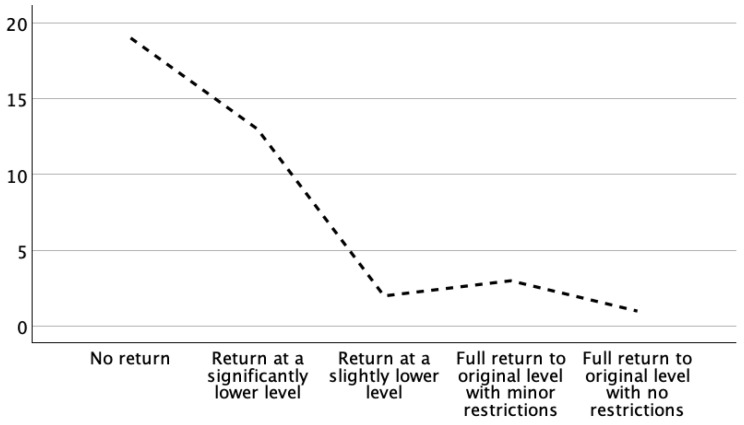
Preoperative evaluation of the return to alpine skiing in patients who suffered a complex proximal tibial fracture (AO/OTA type B or C) from a skiing accident. Only a small proportion of patients expected to return to their original skiing level without any restrictions.

Regarding their return to alpine skiing, 19 (50%) patients stated that they did not expect to be able to ski again, 13 (34.2%) stated that they would be able to ski again at a significantly lower level, 2 (5.3%) patients expected a return to skiing at a slightly lower level, and 4 (10.5%) patients expected that skiing would be possible again at the original level with minor restrictions at most (Figure 1).

### 3.1. Postoperative Fulfillment of Expectations to Return to Alpine Skiing

**Figure 2:**
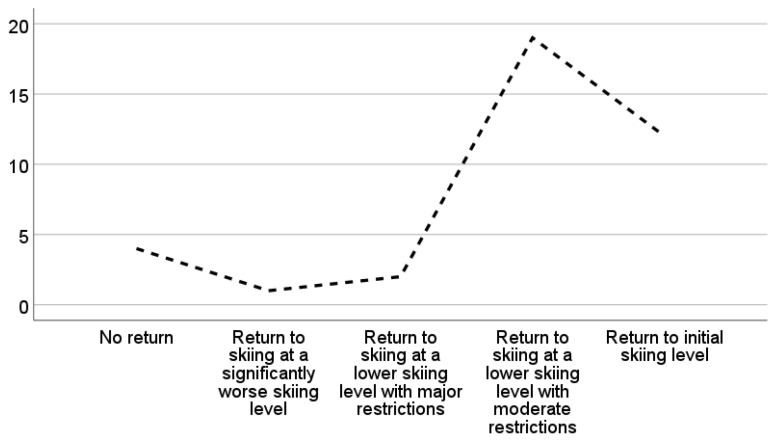
Postoperative evaluation of the return to alpine skiing ≥1 year following open reduction and internal fixation for a complex proximal tibial fracture (AO/OTA type B or C). Of 38 patients, only 12 reached their initial skiing level again postoperatively.

Postoperatively, the return to skiing rate on initial skiing level was 31.6% (n = 12). In total, 19 (50%) patients stated that their initial skiing level was not reached again but they were able to ski with moderate restrictions, 3 (7.9%) patients stated that a return to skiing was possible with major restrictions, and 4 (10.5%) patients stated that no return to skiing was possible (Figure 2).

**Figure 3:**
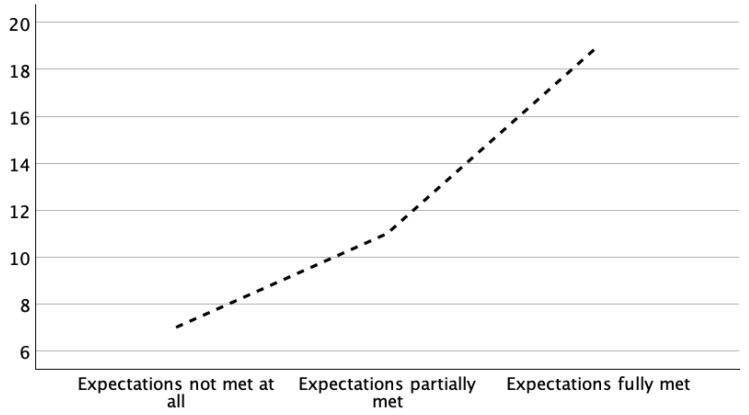
Postoperative fulfillment of patient expectations on their return to alpine skiing following open reduction and internal fixation for a complex proximal tibial fracture (AO/OTA type B or C). Of 38 patients, 19 stated that their initial expectations to return to alpine skiing were fully met ≥ 1 year after surgery.

Regarding the fulfillment of their personal expectations regarding their return to skiing, 19 patients (50%) stated that their expectations were fully met; of these patients, 18 (47.3%) skied regularly (8 male; 11 female). The mean age of these patients was 44.8 years. In total, 11 (29.7%) patients stated that their expectations were partially met, and 7 (18.4%) patients stated that they were not met at all (Figure 3).

The fulfillment of patient expectations did not significantly correlate with the age of the patients. No statistically significant difference could be demonstrated between the two fracture groups, or the gender of the patients (*p* > 0.05).

### 3.2. Postoperative Functional Knee Outcome (KOOS and SF-12)

In the overall study cohort of 38 patients, the mean KOOS for pain was 86.1 (SD 17.1), for symptoms 62.2 (SD 12.9), for activities of daily living (ADL) 90.7 (SD 14.4), for sports 74.2 (SD 25.7), and for quality of life (QOL) 66.6 (SD21.0). No significant differences were found for the various items of the KOOS between the two fracture groups (AO/OTA Type B vs. C) or the gender of the patients (*p* > 0.05). No significant correlation could be demonstrated between age and KOOS.

The mean SF-12 physical component score (PCS) for the total study cohort was 51.7 (SD 9.5). The mean mental component score (MCS) in the total study cohort was 49.9 (SD 7.4). No significant differences could be found between the fracture groups (AO/OTA type B vs. C) or gender (*p* > 0.05). No significant correlation could be demonstrated between age and MCS or PCS.

### 3.3. Evaluation of Postoperative Pain During Skiing

Pain during alpine skiing was evaluated by each patient via NRS. The mean NRS score was 2.3 (SD 2.3). No significant differences could be shown between the two fracture groups (AO/OTA type B vs. C) nor gender (*p* > 0.05). No significant correlation could be demonstrated between age and NRS.

## 4. Discussion

The results of this study provide valuable insights into patient expectations and the postoperative fulfillment of patient expectations in alpine skiers who underwent surgical treatment with open reduction and internal fixation for complex proximal tibial fractures. A key finding is that, preoperatively, patients already expected that alpine skiing would no longer be possible at their initial level. Before surgery, 76% did not prioritize return to skiing as important. Only 10.5% expected to return to alpine skiing with minor restrictions at most. Postoperatively, only 32% of patients returned to their initial skiing level. Fifty percent of patients reported fulfillment of their preoperative expectations to return to skiing. In 18%, preoperative expectations to return to skiing were not fulfilled at all. The type of fracture (AO/OTA type B or C), gender, and/or age was an independent variable. These results indicate an understanding on the part of the patients regarding the severity of their injury.

### 4.1. Preoperative Expectations

Preoperatively, a significant proportion of patients had moderate expectations regarding their ability to return to alpine skiing. Only 5 (13.1%) patients considered it to be “very important” or “important” to return to their initial skiing level, while a larger group (n = 29, 76.3%) did not prioritize this goal as highly. Half of the patients (n = 19) did not expect to ski again, reflecting a degree of realism or pessimism about their recovery prospects. This contrasts with a smaller group (n = 4, 10.5%) who had higher expectations, anticipating a return to skiing with minimal or no restrictions.

### 4.2. Postoperative Fulfillment of Expectations and Functional Outcomes

Postoperatively, a return to the initial skiing level was reached by 31.6% (n = 12) of patients, while 50% (n = 19) could ski but with restrictions, and 10.5% (n = 4) could not return to skiing at all. This distribution highlights a significant portion of patients facing relevant limitations despite surgical intervention.

The KOOS and SF-12 scores provide further context to these outcomes. The mean KOOS values suggest generally good recovery in pain management and activities of daily living (ADL), with pain scores averaging 86.1 and ADL scores at 90.7. However, scores for sports activities (74.2) and quality of life (QOL) (66.6) were lower, indicating areas where patients felt less satisfied.

Pain during skiing, assessed via the Numeric Rating Scale (NRS), averaged 2.3, suggesting that while pain was not severe, it was still present and potentially impacting performance. The absence of significant differences between fracture types, gender, and age groups in KOOS, SF-12, and NRS scores indicates a uniform impact of the injury and subsequent surgery across diverse patient demographics.

The findings of our study align with previous studies which report varied outcomes for patients with tibial plateau fractures [26,27]. Studies have demonstrated that while surgical interventions can restore joint function to a degree, returning to high-impact sports like skiing remains challenging. Return rates to skiing post-surgery vary significantly in the literature, from 53% to 87%, depending on the severity of the injury and the surgical approach used. For instance, Kraus et al. reported that 53% of patients returned to skiing after tibial plateau fractures, while Loibl et al. found return rates as high as 87% in some cases [10,23].

One of the factors why a return to skiing is not possible is the development of post-traumatic osteoarthrosis. Studies have already proven that the risk of secondary OA after proximal tibial fracture is elevated in comparison to a normal population. These patients are at a 3.5- to 5.3-fold higher risk of end-stage post-traumatic OA requiring joint replacement [28,29]. This risk must also be discussed with the patient. Based on the existing literature, the OA rate is very high, depending on the study. Therefore, proximal tibia fracture should not be underestimated as a risk factor for osteoarthritis and the potential need for joint replacement [30,31].

The results of our study and the evaluation of the current literature emphasize that proximal tibia fractures are serious joint injuries. It is therefore even more important to inform patients carefully about possible outcomes. In addition to their inability to participate in high-impact sports, many patients suffer from residual pain and limited knee joint mobility, which also restricts activities of daily living [7,32,33,34]. Limitations such as these may also have a negative impact on mental health. This is also reflected in the results of this study.

### 4.3. Implications for Clinical Practice

Proximal tibial fractures must be considered as severe joint injuries which may lead to restrictions in everyday life and during sporting activities. Thorough patient counseling is important to shape patients’ expectations and to ensure satisfaction with surgical results [35,36,37,38,39,40,41]. Surgeons should ensure that patients have a realistic understanding of the potential outcomes and limitations post-surgery. It is crucial to manage patient expectations and improve satisfaction with surgical results [42,43,44,45]. Besides returning to work, patients’ ability to participate in sporting activities appears to play an important role for a high quality of life and must be taken seriously.

Moreover, these findings highlight the importance of tailored rehabilitation programs that address not just the physical but also the psychological aspects of recovery. Encouraging gradual re-engagement with skiing, possibly with modified techniques or protective measures, could help patients achieve better outcomes. Loibl et al. emphasized the role of comprehensive rehabilitation in improving functional outcomes and facilitating the return to sports [23].

### 4.4. Limitations

This study has several limitations. First, the small sample size (n = 38) limits the generalizability of the findings, especially given the specific patient population involved. Although the prospective approach adds value to the data, a larger cohort would yield more robust insights. Additionally, this study relied primarily on subjective, self-reported data regarding patient expectations and outcomes, which may introduce response bias. Future research should include objective measures, such as testing muscle function, and gait analysis, to provide a more comprehensive evaluation of functional recovery.

## 5. Conclusions

This study contributes to the body of knowledge by highlighting the importance of managing patient expectations and providing realistic forecasts of postoperative outcomes. By aligning surgical goals with patient expectations and emphasizing comprehensive rehabilitation, healthcare providers can enhance both the functional outcomes and overall satisfaction of patients undergoing treatment for complex proximal tibial fractures. Further research with larger sample sizes and diverse cohorts would be beneficial to generalize these findings and refine postoperative care strategies [46,47,48].

## Data Availability

The data presented in this study are available on request from the corresponding author.

## Abbreviations

ADL	Activities of daily living
AO/OTA	Arbeitsgemeinschaft für Osteosynthesefragen
BMI	Body mass index
KOOS	Knee injury and Osteoarthritis Outcome Score
MCS	Mental component score (SF-12)
NRS	Numeric Rating Scale
ORIF	Open reduction and internal fixation
PCS	Physical component score (SF-12)
PROM	Patient-reported outcome measure
QOL	Quality of life
SD	Standard deviation
SF-12	Short-Form 12
